# Frequency histograms of three high‐sensitivity cardiac troponin assays in a reference population

**DOI:** 10.1002/jcla.24432

**Published:** 2022-04-20

**Authors:** Hanwool Cho, Hyunjung Kim, Jehoon Lee, Soo‐Young Kim, Hae Kyung Lee, Hi Jeong Kwon, Yeongsic Kim

**Affiliations:** ^1^ Department of Laboratory Medicine St. Vincent’s Hospital College of Medicine The Catholic University of Korea Seoul Korea; ^2^ Department of Laboratory Medicine Uijeongbu St. Mary’s Hospital College of Medicine The Catholic University of Korea Seoul Korea; ^3^ Department of Laboratory Medicine Eunpyeong St. Mary’s Hospital College of Medicine The Catholic University of Korea Seoul Korea; ^4^ Department of Laboratory Medicine Yeouido St. Mary’s Hospital College of Medicine The Catholic University of Korea Seoul Korea

**Keywords:** 99th percentile upper reference limit, Beckman Coulter Access hs‐cTnI assay, high‐sensitivity cardiac troponin, hs‐cTn

## Abstract

**Background:**

Cardiac troponin (cTn) values above the 99th percentile upper reference limit (URL) indicate myocardial injury. We established 99th percentile URLs for three high‐sensitivity cTn (hs‐cTn) assays (Beckman Coulter Access hs‐cTnI, Abbott STAT hs‐cTnI, and Roche Elecsys hs‐cTnT) using a healthy population in Korea.

**Methods:**

Each cTn value was measured by three assays and analyzed by dividing by gender and age.

**Results:**

The frequency histograms of log‐transformed cTn values for Beckman and Abbott assays exhibited a bell‐shaped distribution. The 99th percentile URLs were 9.8, 17.4, and 17.3 ng/L in the total population; 10.9/9.0, 18.9/17.0, and 18.9/17.7 ng/L in the male/female population (*p* < 0.001 for all three assays); and 11.2/7.2, 19.9/14.5, and 22.7/9.3 ng/L in the older/younger population (*p* < 0.001 for all three assays) for Beckman, Abbott, and Roche assays, respectively.

**Conclusion:**

Among the three assays, bell‐shaped distributions were observed in a frequency histogram of log‐transformed cTn values for healthy population in Beckman and Abbott assays. Also, our findings show that the 99th percentile URLs for cTn levels vary not only by gender but age.

## Graphical abstract

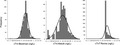

## 1 INTRODUCTION

Cardiac troponin (cTn) is a preferred biomarker in the diagnosis of myocardial infarction and defines a subtype of myocardial infarction.^1^, ^2^ According to the Fourth Universal Definition of Myocardial Infarction (2018),^2^ a cTn level above the 99th percentile upper reference limit (URL) is evidence of myocardial injury and an essential factor in clinical diagnoses of myocardial infarctions. A changing pattern of cTn concentrations obtained during serial measurements above the 99th percentile URL is required to differentiate acute from chronic increases.^2^ Discrimination of small amounts of change in cTn values, therefore, requires considerable analytical sensitivity and precision, particularly at low concentrations. The International Federation of Clinical Chemistry and Laboratory Medicine (IFCC) proposed that high‐sensitivity cTn (hs‐cTn) assays should have an imprecision of no more than 10% of the total coefficient of variation at a concentration of the 99th percentile URL in a healthy population, and the proportion of measurable values at or above the limit of detection (LOD) must be at least 50% in a healthy population.^3^

cTn levels differ by age, gender, and ethnicity, and substantial differences in cTn levels may be observed between assays due to the lack of standardization in cTn measurement. The analytical performance and the 99th percentile URLs of hs‐cTn assays were evaluated in previous studies with a large number of healthy individuals, and the results showed the difference in the 99th percentile URLs by age, sex, and assays.^4^, ^5^, ^6^

Here, we measured cTn values in a healthy population using three hs‐cTn assays, Beckman Coulter Access high‐sensitivity cardiac troponin I (hs‐cTnI), Abbott STAT hs‐cTnI, and Roche cobas Elecsys high‐sensitivity cardiac troponin T (hs‐cTnT) assays. The frequency histograms of the cTn levels from each assay were visually inspected for differences in imprecision at low concentrations of cTn values in the three assays. We established the 99th percentile URL of cTn using these three hs‐cTn assays in a Korean population, and the differences in 99th percentile URLs by gender and age were evaluated.

## 2 MATERIALS AND METHODS

### 2.1 High‐sensitivity cTn analyses

Serum remnants were analyzed from specimens collected for routine chemistry testing at St. Vincent’s Hospital, Suwon, Republic of Korea, from September to November of 2019. All samples were centrifuged at 2000 ***g*** for 10 min and stored at −70°C within 2 h after sample collection and thawed once before analysis. The study protocols were approved by the Institutional Review Board of St. Vincent’s Hospital. This study was conducted in accordance with the Declaration of Helsinki.

We measured cTn concentrations of serum samples using Beckman hs‐cTnI, Abbott hs‐cTnI, and Roche hs‐cTnT on UniCel DxI800 (Beckman Coulter), ARCHITECT i2000SR (Abbott), and cobas e411 (Roche) analyzer platforms, respectively. All are two‐step chemiluminescent immunoassays. All analysis was performed on one analytical batch in each assay. The claimed LODs by each manufacturer were 2.3, 1.9, and 5.0 ng/L for the Beckman hs‐cTnI, Abbott hs‐cTnI, and Roche hs‐cTnT assays, respectively. The LOD of the Beckman hs‐cTnI assay was estimated according to the Clinical and Laboratory Standards Institute EP17‐A.^7^ The LOD of Abbott hs‐cTnI was taken from Apple et al.^8^ According to the manufacturers, the repeatability and within‐laboratory imprecision coefficients of variation were 4% and 7%, and 4% and 4% for Beckman hs‐cTnI and Abbott hs‐cTnI, respectively, and the repeatability and between‐run imprecision coefficients of variation were 3% and 3% for Roche hs‐cTnT assay.

### 2.2 Establishment of the 99th percentile URL

Patients involved in the establishment of the 99th percentile URL were at least 20 years of age; had no cardiovascular disease, diabetes mellitus, history of cardiovascular disease, and no cardiac medication use according to a review of medical records and questionnaires; and had an estimated glomerular filtration rate (eGFR) ≥60 ml/min/1.73 m^2^ according to the Chronic Kidney Disease Epidemiology Collaboration equation.^9^ We also established the 99th percentile URLs by using eGFR exclusion criteria of 90 ml/min/1.73 m^2^. The 99th percentile URLs were established according to gender and age (≤51 years and >51 years). A nonparametric statistical method with 5000 bootstrap for establishing the 99th percentile URLs^10^ and the Reed‐Dixon method for outlier exclusion^11^, ^12^ were performed according to IFCC recommendations. The log‐transformed values were used to establish and exclude outliers. As the Roche hs‐cTnT assay show results of 3 ng/L or less as ≤3 ng/L, the 99th percentile URL was established by treating the values of ≤3 ng/L as 3 ng/L.

### 2.3 Statistical analysis

Mann–Whitney tests were used to compare continuous variables, Shapiro–Wilk tests were used to test normality of distribution of cTn value, and multiple regression analyses were used to estimate relationship between cTn values and independent variables in MedCalc, and a two‐tailed *p* value of ≤0.05 was considered statistically significant. The 99th percentile URLs were established in MedCalc 20.008 (MedCalc Software) and Analyse‐it v.5.81 (Analyse‐it Software, Ltd.).

## 3 RESULTS

### 3.1 Measurable values of cTn in the healthy population

The characteristics of the study population are shown in Table 1. The measurable cTn values of the three assays are shown in Table 2. The measurable cTn values at or above the manufacturer’s claimed LOD were 46.5% (290/623), 40.1% (250/623), and 18.5% (115/623) for the Beckman hs‐cTnI, Abbott hs‐cTnI, and Roche hs‐cTnT assays, respectively. The estimates for LOD were 0.6 and 1.2 ng/L for the Beckman hs‐cTnI and Abbott hs‐cTnI assays, respectively. The measurable cTn values at or above the estimates for LOD were 99.5% (620/623) and 64.7% (403/623) for the Beckman hs‐cTnI and Abbott hs‐cTnI assays, respectively.

**TABLE 1:** Characteristics of study population

	Population with eGFR ≥ 60 ml/min/1.73 m^2^						Population with eGFR ≥ 90 ml/min/1.73 m^2^					
	Male (*N* = 307)	Female (*N* = 316)	*p*‐Value	≤51 years (*N* = 315)	>51 years (*N* = 308)	*p*‐Value	Male (*N* = 219)	Female (*N* = 279)	*p*‐Value	≤51 years (*N* = 297)	>51 years (*N* = 201)	*p*‐Value
Age, years (median, IQR)	53 (44–63)	50 (41–60)	0.002				49 (41–56)	48 (40–57)	0.416			
Male, *N* (%)				143 (45.4)	164 (53.2)	0.050				132 (44.4)	87 (43.3)	0.798
HbA1c^a^, % (median, IQR)	5.3 (5.2–5.5)	5.3 (5.1–5.5)	0.112	5.3 (5.1–5.4)	5.5 (5.2–5.7)	<0.001	5.3 (5.2–5.5)	5.3 (5.1–5.5)	0.482	5.3 (5.1–5.4)	5.4 (5.2–5.7)	<0.001
eGFR, ml/min/1.73 m^2^ (median, IQR)	99.0 (88.2–107.9)	105.5 (97.9–113.9)	<0.001	110.7 (104.9–116.4)	94.7 (85.6–100.5)	<0.001	104.9 (97.2–110.2)	107.8 (100.2–114.0)	<0.001	99.0 (94.9–103.3)	111.0 (106.0–116.9)	<0.001
GFR category^b^, *N* (%)												
G1	219 (71.3)	279 (88.3)	<0.001	297 (94.3)	201 (65.3)	<0.001						
G2	88 (28.7)	37 (11.7)		18 (5.7)	107 (34.7)							

^a^ 289 of samples (147 male and 142 female, 189 young and 100 old) and 247 of samples (116 male and 131 female, 176 young and 71 old) had accompanying HbA1c results in the population with eGFR ≥60 and eGFR ≥90 ml/min/1.73 m^2^, respectively.

^b^ G1 and G2 in GFR categories are GFR ≥90 ml/min/1.73 m^2^ and 60–89 ml/min/1.73 m^2^.

**TABLE 2:** The limit of detection and the percent measurable value of cTn for the assays in a healthy population (*N* = 623)

Assay	LOD, ng/L	Measurable value	% measurable value
Beckman Coulter hs‐cTnI	0.6^a^	620	99.5
	2.3^b^	290	46.5
Abbott hs‐cTnI	1.2^c^	403	64.7
	1.9^b^	250	40.1
Roche hs‐cTnT	5.0^b^	115	18.5

Abbreviation: LOD, limit of detection.

^a^ Estimate for LOD.

^b^ Claimed LOD by manufacturer.

^c^ Taken from Apple et al.^8^

### 3.2 Distribution of cardiac troponin levels in the healthy population

**FIGURE 1:**
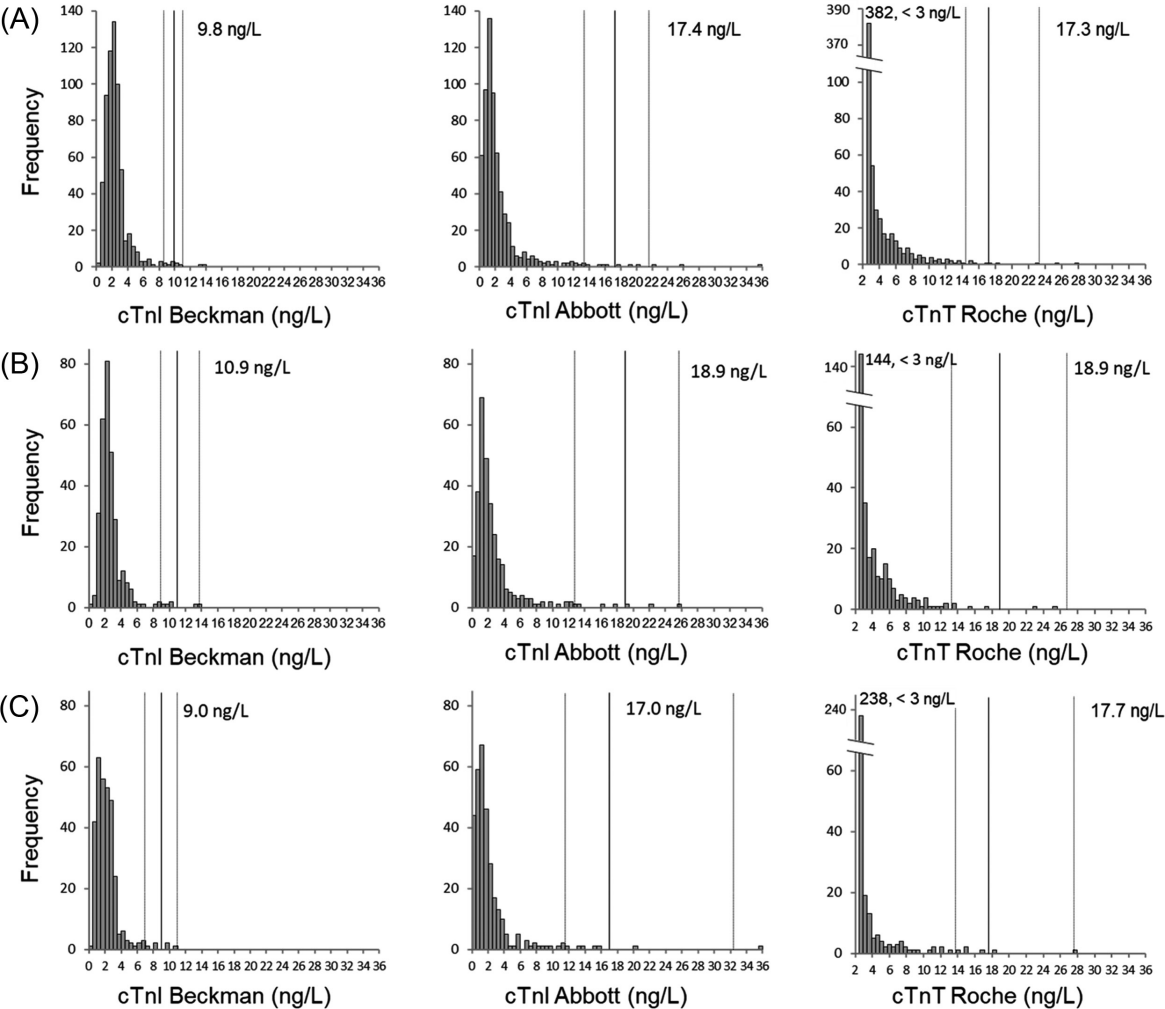
Frequency histograms of specimens from (A) an all‐healthy population, (B) the male population, and (C) the female population measured for cTn by three assays. The black and gray lines represent the 99th percentile URLs and 90% CI, respectively

**FIGURE 2:**
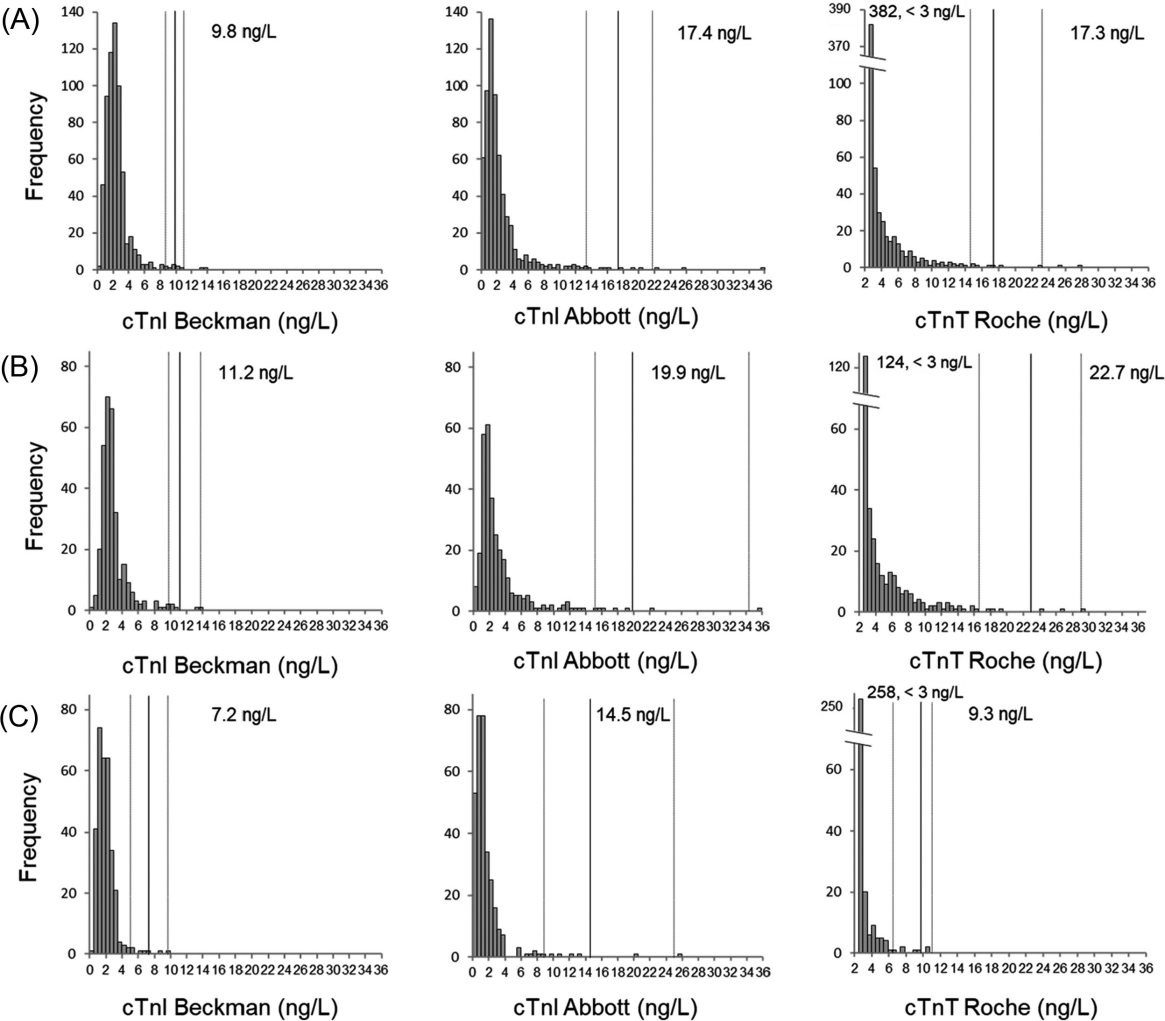
Frequency histograms of specimens from (A) an all‐healthy population, (B) the older population (>51 years), and (C) the younger population (≤51 years) measured for cTn by three assays. The black and gray lines represent the 99th percentile URLs and 90% CI, respectively

**FIGURE 3:**
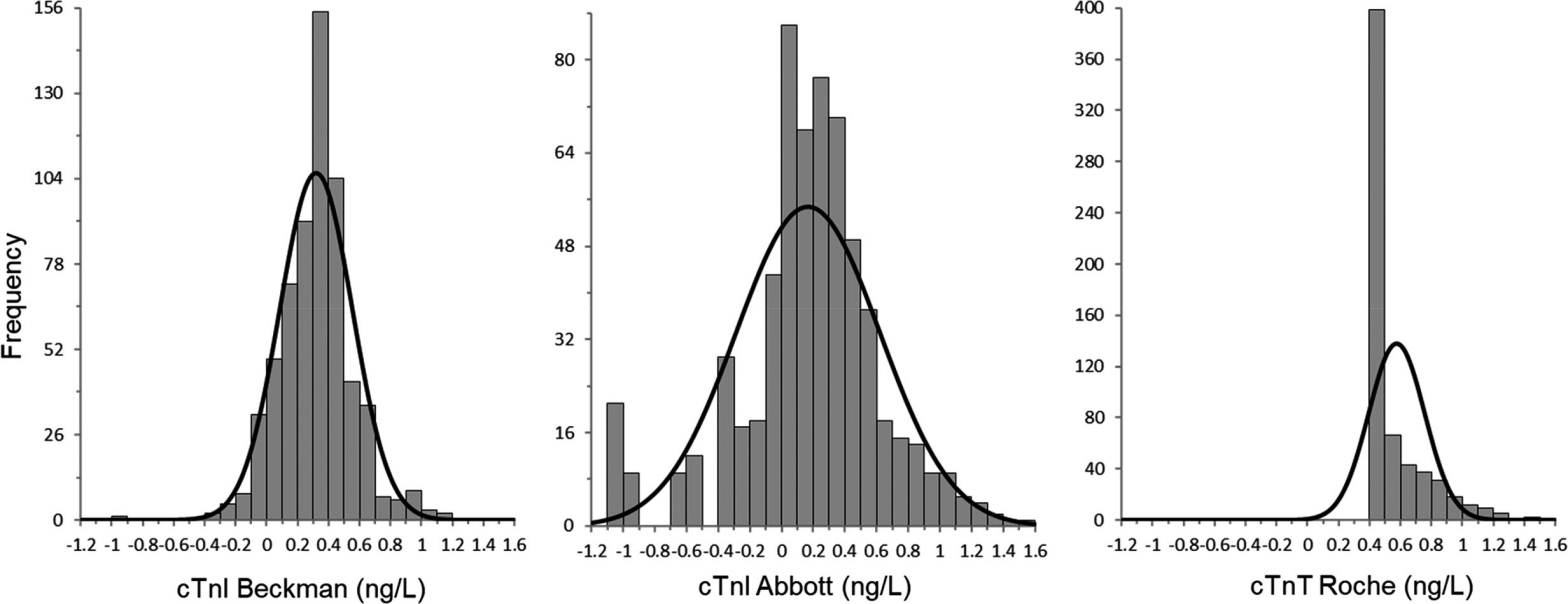
Frequency histograms of the log‐transformed cTn values by three assays

Frequency histograms of cTn levels are shown in Figures 1 and 2, and frequency histograms of the log‐transformed cTn levels are shown in Figure 3. The distributions of the log‐transformed cTn values in all three assays were not normal (*p*‐value < 0.001 for all three assays). Although the distribution of the log‐transformed values of Beckman hs‐cTnI and Abbott hs‐cTnI showed near‐Gaussian distribution (*W* = 0.9742 and 0.9613, respectively), the distribution of the log‐transformed values of Roche hs‐cTnT showed right‐skewed distribution (*W* = 0.6398) (Figure 3).

As shown in the frequency histograms generated by gender and age, the median value shifted to the right in the male population compared to the female population and also in the older population compared to the younger population. The median cTn values for the Beckman hs‐cTnI, Abbott hs‐cTnI, and Roche hs‐cTnT assays were 2.3 ng/L (interquartile range [IQR] 1.8–3.0), 1.8 ng/L (IQR 1.1–2.9), and 3.1 ng/L (IQR 3.0–5.2) in the male population and 1.9 ng/L (IQR 1.2–2.6), 1.3 ng/L (IQR 0.8–2.4), and 3.0 ng/L (IQR 3.0–3.0) in the female population, respectively (*p* < 0.001 for all three assays). The median cTn values for the Beckman hs‐cTnI, Abbott hs‐cTnI, and Roche hs‐cTnT assays were 2.5 ng/L (IQR 1.9–3.1), 2.1 ng/L (IQR 1.3–3.5), and 3.5 ng/L (IQR 3.0–6.0) in the older population and 1.8 ng/L (IQR 1.2–2.4), 1.0 ng/L (IQR 0.6–1.8), and 3.0 ng/L (IQR 3.0–3.0) in the younger population, respectively (*p* < 0.001 for all three assays).

### 3.3 99th percentile URL of cTn using assays in the Korean population

The 99th percentile URL for cTn levels using a nonparametric method with an outlier exclusion and the Reed‐Dixon method is shown in Table 3. When all samples were analyzed, the 99th percentile URLs were 9.8 ng/L (90% confidence interval [CI] 8.6–10.8), 17.4 ng/L (90% CI 13.3–21.5), and 17.3 ng/L (90% CI 14.3–23.1) using the Beckman hs‐cTnI, Abbott hs‐cTnI, and Roche hs‐cTnT assays, respectively.

**TABLE 3:** The established 99th percentile URLs in healthy population using three assays

Assay	Established 99th percentile URL, ng/L (90% CI)									
	Population with eGFR ≥60 ml/min/1.73 m^2^					Population with eGFR ≥90 ml/min/1.73 m^2^				
	Total (*N* = 623)	Male (*N* = 307)	Female (*N* = 316)	≤51 years (*N* = 315)	>51 years (*N* = 308)	Total (*N* = 498)	Male (*N* = 219)	Female (*N* = 279)	≤51 years (*N* = 297)	>51 years (*N* = 201)
Beckman Coulter hs‐cTnI	9.8 (8.6‒10.8)	10.9^a^ (8.7‒13.6)	9.0 (6.9‒10.7)	7.2 (5.0‒9.6)	11.2^a^ (9.7‒13.6)	8.2 (5.2‒10.4)	8.8 (5.6‒10.4)	7.7 (5.0‒10.9)	6.5 (4.9‒8.7)	9.9 (5.7‒10.9)
Abbott hs‐cTnI	17.4 (13.3‒21.5)	18.9 (12.6‒25.6)	17.0 (11.5‒32.2)	14.5 (8.7‒25.0)	19.9 (15.1‒34.3)	15.8 (11.5‒22.0)	15.7 (11.0‒22.0)	18.0 (10.7‒35.5)	12.1 (8.1‒20.1)	22.8 (15.1‒35.5)
Roche hs‐cTnT	17.3 (14.3‒23.1)	18.9 (13.4‒26.6)	17.7 (13.8‒27.6)	9.3 (6.5‒10.9)	22.7 (16.4‒29.1)	12.6 (9.2‒18.3)	12.7 (9.5‒18.3)	14.3 (7.9‒29.3)	8.7 (6.5‒10.9)	19.8 (11.7‒29.3)

Abbreviations: CI, confidence interval; URL, upper reference limit.

^a^One value was excluded as an outlier by the Reed‐Dixon method.

The 99th percentile URLs for cTn were higher in male samples than in female samples from all three assays. The 99th percentile URLs in male and female samples were 10.9 ng/L (90% CI 8.7–13.6) and 9.0 ng/L (90% CI 6.9–10.7) for the Beckman hs‐cTnI assay (*p* < 0.001), 18.9 ng/L (90% CI 12.6–25.6) and 17.0 ng/L (90% CI 11.5–32.2) for the Abbott hs‐cTnI assay (*p* < 0.001), and 18.9 ng/L (90% CI 13.4–26.6) and 17.7 ng/L (90% CI 13.8–27.6) for the Roche hs‐cTnT assay (*p* < 0.001). The 99th percentile URLs were higher in the older population in all three assays. The 99th percentile URLs in the older and younger populations were 11.2 ng/L (90% CI 9.7–13.6) and 7.2 ng/L (90% CI 5.0–9.6) for the Beckman hs‐cTnI assay (*p* < 0.001), 19.9 ng/L (90% CI 15.1–34.3) and 14.5 ng/L (90% CI 8.7–25.0) for the Abbott hs‐cTnI assay (*p* < 0.001), and 22.7 ng/L (90% CI 16.4‒29.1) and 9.3 ng/L (90% CI 6.5‒10.9) for the Roche hs‐cTnT assay (*p* < 0.001). In the multiple regression analysis, cTn values were increased with age for three assays (*p* < 0.001 for all three assays), and cTn values measured by Beckman and Roche assays showed association with gender (*p* = 0.003 and 0.048, respectively), but cTn values measured by Abbott assay showed no statistically significant association with gender (*p* = 0.264).

As eGFR showed significant differences by gender and age in the study population (*p* < 0.001 for gender and age), the URLs were obtained from the population with ≥90 ml/min/1.73 m^2^ (Table 3). The 99th percentile URLs were lower than those obtained from samples with eGFR ≥60 ml/min/1.73 m^2^. Although the 99th percentile URL was higher in male than in female population for the Beckman hs‐cTnI assay, the 99th percentile URLs were higher in female than in male population for the Abbott hs‐cTnI assay and the Roche hs‐cTnT assay. The 99th percentile URLs were higher in older than in younger population for all three assays.

## 4 DISCUSSION

The Beckman and the Abbott hs‐cTnI assays measured cTn values above their LOD in 99.5% and 64.7% of healthy individuals, and their frequency histograms of the log‐transformed cTn values showed a bell‐shaped distribution due to their robust detection capabilities. Previous studies reported the excellent analytical sensitivity of the Beckman hs‐cTnI assay and higher measurable values above the LOD in healthy populations.^13^, ^14^, ^15^ Measurable values of the Abbott hs‐cTnI assay were obtained in 64.7% of a healthy population when the estimate for the LOD was applied. For the Roche hs‐cTnT assay, only 18.5% of the healthy population had levels above its LOD, and 61.3% showed a cTn value as “below the LOB” (<3 ng/L). This is line with previous studies using the Abbott hs‐cTnI^16^, ^17^, ^18^ and Roche hs‐cTnT assays.^18^, ^19^, ^20^ As the Roche hs‐cTnT assays could not produce accurate cTn values in a significant number of the healthy population, the frequency histograms of the log‐transformed cTn values using the assay showed a right‐skewed distribution in the healthy population. This difference in histograms well indicates the difference in detection capability between the Roche hs‐cTnT assay and other two assays.

Sex‐specific URLs of hs‐cTn are recommended in the IFCC guidelines, but using age‐specific URLs remains controversial.^3^, ^21^ There is a concern that the URL will increase because the older “healthy” population is likely to include individuals with unrecognized comorbidities.^22^ In this study, the distribution curve in older subjects not only showed a longer right tail but also shifted to the right compared with data from younger subjects. This implies that the URL is higher at older ages because cTn values increase with age, not because of the higher proportion of individuals with subclinical cardiovascular disease. The URLs obtained from the population with eGFR ≥90 ml/min/1.73 m^2^ were lower than from the total population (eGFR ≥60 ml/min/1.73 m^2^) in all three assays. In the population with eGFR ≥90 ml/min/1.73 m^2^, although the URLs in older population were still higher than in younger population for all three assays, the URL in male population was higher than in female population only for the Beckman hs‐cTnI assay. These results may be explained by the no significant difference in median age between genders and the small number of individuals less than 300 in the population with eGFR ≥90 ml/min/1.73 m^2^, unlike the total population. However, the difference in URLs between age groups is still clear even though the effects of gender are decreased. In addition, multiple regression analysis showed that cTn values correlated with age in all three assays. In line with this study, Gore et al.^23^ proposed age‐ and sex‐specific URLs for the cTnT assay. These results indicate that if a uniform URL is applied without considering age, the false‐positive rate in older subjects may increase. However, further studies about the clinical utility of an age‐specific URL are required.

Cardiomyocytes are physiologically remodeled, and small amounts of cardiac troponin are released from apoptotic cells by physiological remodeling in normal healthy adults.^24^ Cardiomyocyte remodeling, which may be affected by factors such as age and exercise, may result in different cTn levels depending on age and sex in healthy populations.

The troponin values of at least 300 individuals per group are needed to establish URLs by gender and age to ensure a 95% probability that at least 99% of the population will have values lower than the highest observed troponin values.^3^, ^10^, ^25^ In this study, the troponin values of about 300 individuals per group were used to established URLs according to gender and age. Although the URLs of previous studies using larger populations than this study were different from the URLs of this study,^5^, ^6^, ^14^ all studies showed difference in URLs by gender and age. The difference in URLs can be interpreted due to differences in demographic characteristics such as ethnicity and age of the population. However, since this study obtained URLs using a smaller number of individuals than previous studies, caution is required in this interpretation.

Although it is recommended to exclude individuals using the surrogate biomarker concentrations including NT‐proBNP, HbA1c, and eGFR,^3^ BNP levels were not included in the exclusion criteria, and only eGFR and HbA1c were included in this study. The surrogate biomarker, NT‐proBNP, was recommended for definition of reference population^10^, ^25^ to exclude individuals with asymptomatic myocardial injury. In a previous study to establish the 99th percentile URL of cTnI by including NT‐proBNP in the definition of a healthy population, the 99th percentile URLs showed a significant difference between the overall population and the population with NT‐proBNP in the normal range.^16^ However, the use of surrogate biomarkers for selecting healthy individuals is controversial.^26^, ^27^, ^28^ This may limit the interpretation of the study results because the individuals with silent pathology may be included in this study.

## 5 CONCLUSIONS

The Beckman and Abbott assays detected cTn values in more than 50% of healthy population, and the log‐transformed cTn values of these two assays showed bell‐shaped distributions in a frequency histogram for healthy population. Our findings also demonstrate that the 99th percentile URLs of cTnI and cTnT vary by both gender and age. Using age‐ and sex‐specific URLs of cTn could therefore reduce false‐positive rates and reduce unnecessary diagnostic procedures for AMI.

## CONFLICT OF INTEREST

None declared.

## PATIENT CONSENT STATEMENT

This study was approved by the Institutional Review Board of St. Vincent's Hospital and was granted a waiver of informed consent.

## Data Availability

The data that support the findings of this study are not publicly available due to the privacy of research participants but are available from the corresponding author upon reasonable request.
